# Impact of Tat Genetic Variation on HIV-1 Disease

**DOI:** 10.1155/2012/123605

**Published:** 2012-07-30

**Authors:** Luna Li, Satinder Dahiya, Sandhya Kortagere, Benjamas Aiamkitsumrit, David Cunningham, Vanessa Pirrone, Michael R. Nonnemacher, Brian Wigdahl

**Affiliations:** ^1^Department of Microbiology and Immunology, Drexel University College of Medicine, 245 N. 15th Street, MS no. 1013A, Philadelphia, PA 19102, USA; ^2^Center for Molecular Virology and Translational Neuroscience, Institute for Molecular Medicine and Infectious Disease, Drexel University College of Medicine, 245 N. 15th Street, MS no. 1013A, Philadelphia, PA 19102, USA

## Abstract

The human immunodeficiency virus type 1 (HIV-1) promoter or long-terminal repeat (LTR) regulates viral gene expression by interacting with multiple viral and host factors. The viral transactivator protein Tat plays an important role in transcriptional activation of HIV-1 gene expression. Functional domains of Tat and its interaction with transactivation response element RNA and cellular transcription factors have been examined. Genetic variation within *tat* of different HIV-1 subtypes has been shown to affect the interaction of the viral transactivator with cellular and/or viral proteins, influencing the overall level of transcriptional activation as well as its action as a neurotoxic protein. Consequently, the genetic variability within *tat* may impact the molecular architecture of functional domains of the Tat protein that may impact HIV pathogenesis and disease. Tat as a therapeutic target for anti-HIV drugs has also been discussed.

## 1. Introduction

The human immunodeficiency virus type 1 (HIV-1) is the causative agent of acquired immunodeficiency syndrome (AIDS). The HIV-1 genome is about 9.8 kb in length, including two viral long-terminal repeats (LTRs) located at both ends when integrated into the host genome. The genome also includes genes that encode for the structural proteins ([Gag], [Pol], and [Env]), regulatory proteins (Tat and [Rev]), and accessory proteins ([Vpu], [Vpr], [Vif], and [Nef]). The HIV-1 transactivator of transcription (Tat) protein is an early regulatory protein containing from 86 to 106 amino acids in length with a molecular weight of approximately 14 to 16 kDa. Tat is a multifunctional protein that has been proposed to contribute to several pathological consequences of HIV-1 infection. Tat not only plays an important role in viral transcription and replication, it is also capable of inducing the expression of a variety of cellular genes as well as acting as a neurotoxic protein. In this review, the functions of Tat and molecular diversity in Tat are addressed. Moreover, the interaction of Tat with the viral LTR and cellular factors are documented and discussed. Because of its pivotal role in viral replication and disease pathogenesis, Tat and the cellular pathways targeted by Tat could be potential targets for new anti-HIV drugs. Therapeutic strategies that have focused on this topic are also reviewed.

## 2. Functional Domains of the Transactivator ****Protein Tat 

**Figure 1:**
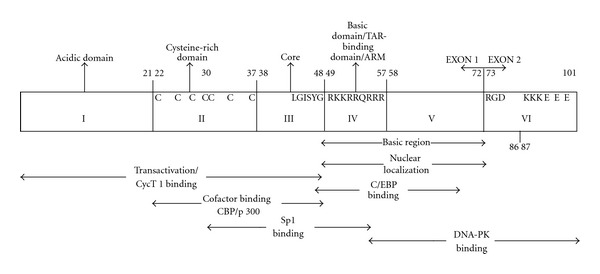
Schematic representation of HIV-1 Tat with locations of the six main domains indicated. Within each domain, important amino acid residues are designated. In addition, known functions of the domains or interactions with the protein involved in transcription are also highlighted.

Tat is a 14 to 16 kDa nuclear protein. It is a multifunctional protein, which is essential for the productive and processive transcription driven from the HIV-1 LTR promoter, and is required for overall productive viral replication [1, 2]. It is a 101-amino acid protein encoded by two exons: the first exon encodes amino acids 1 to 72; the second encodes residues from 73 to 101 (Figure 1) [3]. Most clinical HIV-1 isolates of Tat include 101 amino acids, whereas a few isolates contain from 86 to 106 amino acids, with the second exon coding from 14 to 34 residues at the C terminus of the protein [4]. The HIV-1 IIIB Tat used in many *in vitro* experiments contains 86 amino acids, corresponding to HIV-1 (strain BRU) or a closely related sequence from the HXB2 HIV-1 infectious molecular clone [5, 6]. This 86-amino acid configuration of Tat is the most frequently used form for laboratory investigations; however, it must be noted that it represents a truncated protein when compared to Tat from many clinical isolates. Several studies have established that HIV-1 Tat maintains the 101-amino acid composition as previously reviewed [7]. The more truncated 86-amino acid version of Tat appears to be functional [4], but functions like modulation of host cell cytoskeleton modifications [8] and perhaps optimal replication in cells of the monocyte-macrophage lineage have been attributed to the second exon. Also, the fact that most clinical isolates preserve the full 101-amino acid form is indicative of the functional relevance of the second exon in an *in vivo* setting.

Tat has been divided into six different functional domains (Figure 1) [3, 4, 9]. The N-terminal domain (residues 1–21, also known as the acidic domain) is a proline-rich region containing a conserved tryptophan residue and a number of acidic amino acids. This region is able to form an *α*-helix and is tolerant of numerous single-residue changes without severe compromise in protein function. The second domain (residues 21–37, also referred to as the cysteine-rich domain) contains a highly conserved cysteine-rich tract including seven cysteines at positions 22, 25, 27, 30, 31, 34, and 37, four of which are responsible for the formation of disulfide bridges; changes in any one of six of the seven cysteines significantly affect Tat function [10]. The third domain (residues 38–48) has a hydrophobic core sequence: _43_LGISYG_48_. The first three domains (amino acid 1–48) comprise the minimal region for Tat transactivation capability. Within this region, genetic variation resulting in changes in amino acids from 1 to 21 is typically tolerated; however, changes in residues from 22 to 40 are deleterious with respect to transactivation. The fourth domain (residues 49–57, the basic domain) is a positively charged region composed of a well-conserved _49_RKKRRQRRR_57_ motif, also known as the arginine-rich motif, or transactivation response element- (TAR) binding domain. This region is necessary for Tat nuclear localization, binding to the HIV-1 leader RNA TAR [11–13], and uptaken by other cells [14, 15]. Studies have also demonstrated that Tat utilizes the basic domain residues from 48 to 60 for the functional internalization into cells [16, 17]. The fifth domain (residues 58–72) is a glutamine-rich region shown to exhibit the greatest degree of genetic variability. The fourth and fifth domains together (residues 49–72) are referred to as the basic region. The sixth domain (amino acid 73–101) encoded by the second exon has been less well characterized but may contribute to viral infectivity and binding to cell-surface integrins [18–20]. Two short motifs have been identified in the C terminus of Tat: the RGD (arg-gly-asp) motif, which is a ligand for several integrins, and the highly conserved ESKKKVE motif, which may be related to optimal HIV-1 replication *in vivo* [18, 21]. Although the transactivation domain has been localized to Tat exon I, Tat exon II also plays a role in kappa-light-chain-enhancer of activated B cell-(NF-*κ*B) dependent control of HIV-1 transcription in T cells [22]. The glutamic acid residues 92, 94, and 96 or lysine residues 88, 89, and 90 within the Tat exon II exhibit a critical role in activating NF-*κ*B, transactivating the HIV-1 LTR, and enhancing HIV-1 replication in T cells.

## 3. Basal and Stimulated Transactivation Driven by the HIV-1 LTR

**Figure 2:**
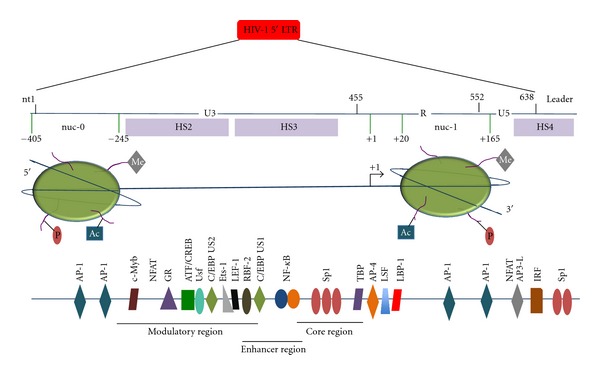
Molecular architecture of the HIV-1 long-terminal repeat. The viral promoter, the long-terminal repeat (LTR), can be divided into the U3, R, and U5 regions. Upon integration, the LTR presents stretches of DNase I hypersensitivity sites (shown as HS2, HS3, and HS4) as a result of the well-defined, conversed positioning of the two nucleosomes; nuc-0 and nuc-1. This architecture results in exposure of stretches of DNA extremely rich in transcription factor-binding sites that include different regulatory proteins in the process of HIV-1 transcription. These factors respond to various extracellular and intracellular stimuli, resulting in upregulation/downregulation of specific downstream transcription factors that act via binding to their respective binding sites in the LTR. Also, positioning of nuc-1 is crucial because it is present immediately downstream of the start site (+1); this nucleosome needs to be remodeled for active processive transcription to ensue from the LTR. Moreover, modifications like acetylation, phosphorylation, and methylation of histone tails regulate LTR-directed transcription. The HIV-1 Tat protein regulates the chromatin environment via interactions with several components including methyltransferases, acetyltransferases, and a number of transcription factors in addition to binding to the TAR element in nascent HIV-1 RNA.

The HIV-1 LTRs are generated during the process of reverse transcription and located on each end of the proviral DNA when the provirus is integrated into the host genome. The LTR is approximately 640 base pairs in length and divided into the unique 5′ (U5) and 3′ (U3) regions as well as the repeat region (Figure 2). LTR sequences include four functional regulatory regions with respect to the control of HIV-1 transcription: TAR element, core promoter, enhancer region, and modulatory region [47]. A multitude of HIV-1 promoter regulatory elements are located within the U3 region of the 5′ LTR and drive the production of HIV-1 mRNA that codes for proteins involved in regulating viral replication as well as the assembly and release of infectious progeny virus. The core region of the LTR is composed of the TATAA box, which is located 29–24 nucleotides upstream of the transcriptional start site, and specificity protein (Sp) binding sites, which are three tandem GC-rich binding sites (−45 to −77) interacting with transcription factors Sp1 through Sp4. The TATAA box binds TATAA-binding protein in association with a number of other proteins that comprise the RNA polymerase II (pol II) transcription complex for transcription initiation and elongation [48–50]. The enhancer element is primarily composed of two copies of 10-base pair binding sites for NF-*κ*Bs and related proteins [51]. The modulatory region, which is in the 5′ end of the U3 region, contains binding sites for many factors, including CCAAT/enhancer-binding protein (C/EBP) factors [52], activating transcription factor/cyclic AMP response element-binding protein (ATF/CREB) [53], nuclear factor of activated T cells (NFAT) [54], and a number of other proteins, depending on cell phenotype, differentiation status, and state of activation (Figure 2) [55–58].

The integrated proviral DNA interfaces with the normal molecular architecture of the host chromatin, which is assembled into nucleosomes. Each nucleosome contains a protein core made of eight histone molecules (H2A, H2B, H3, and H4) and 146 nucleotide-long double-stranded DNA wrapped around it [59]. Independent of the integration site, two nucleosomes (designated nuc-0 and nuc-1) are precisely organized on the HIV-1 viral promoter DNA (Figure 2) [60, 61]. In a transcriptionally quiescent state, nuc-0 is positioned at nucleotide from −405 to −245 relative to the transcriptional start site, and nuc-1 is positioned at nucleotide from +20 to +165 relative to the transcriptional start site (Figure 2). These wrapped regions define two open nucleosome-free regions in the viral DNA, extending from −244 to +19 and +166 to +256 relative to the transcription start site (Figure 2). These open regions include the HIV-1 LTR modulatory, enhancer/core region, transcription factor-binding sites for AP3-L, Sp1 [60], and upstream regulatory factor (USF) [62], and a region overlapping the primer-binding site immediately downstream of the 5′ LTR [63]. It has been proposed and shown that displacement of nuc-1 is a prerequisite for HIV-1 transcription as observed in response to T cell activation stimuli [60]. Conformation of the nucleosomes is modulated in two ways: (1) posttranslational modifications of N-terminal tails of histones, namely acetylation, phosphorylation, and methylation through factors like histone acetyl transferases (HATs), histone deacetylases (HDACs), histone methyltransferases (HMTs), and kinases and (2) ATP-dependent chromatin remodeling complexes such as P300/CBP-associated factor (PCAF) and the SWI/SNF family [64]. 

**Figure 3:**
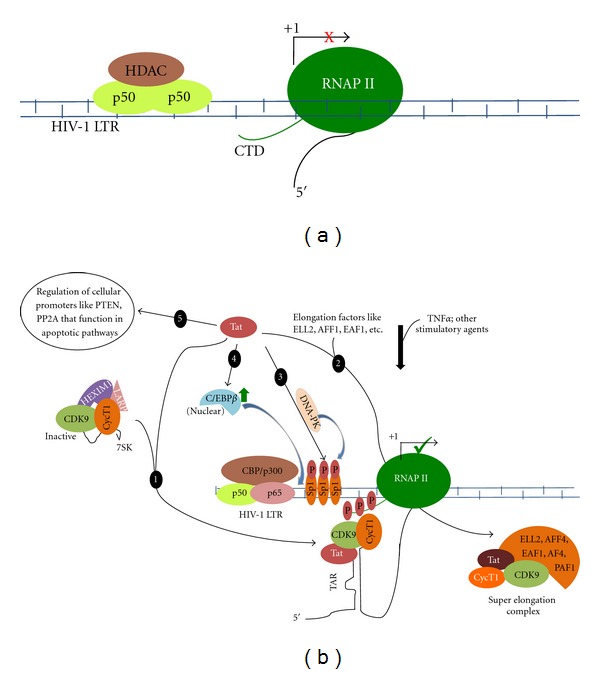
Functions of Tat. Tat plays a crucial role in synthesis of full-length HIV-1 mRNA transcripts. In the absence of Tat, the viral promoter remains latent with NF-*κ*B (p50 homodimers) and histone deacetylase complexes (HDACs) maintaining a repressive chromatin environment by deacetylating the histones (a). Thus, RNA polymerase-(RNAPII) driven transcription is not progressive- and full-length HIV-1 transcripts are not synthesized. (b) Under a cellular stimulation environment, the latent state is overcome by recruitment of CBP/p300 complex to the viral promoter, the LTR. The Tat protein translocates to the nucleus and triggers a release of P-TEFb (CDK9/CycT1) from an inactive complex with HEXIM1, LARP, and 7SK RNA (1). The active P-TEFb in complex with Tat then interacts with the stem-loop structure (TAR) in the nascent HIV-1 mRNA's 5′ end. This event triggers the phosphorylation of the C-terminal domain (CTD) of RNAPII by CDK9, resulting in productive transcription from the LTR (b). Tat also participates in formation of a “super elongation complex” with factors including ELL2, AFF1, EAF1, and others, which also aids in synthesis of full-length HIV-1 transcripts from the relieved template (2). Tat also recruits another kinase DNA-PK in a ternary complex with Sp1 transcription factor at the LTR (3). This results in phosphorylation of Sp1 and activation of Sp1-mediated LTR-directed transcription. Tat also interacts with C/EBP*β* and triggers an increase in the nuclear levels of C/EBP*β*, again indirectly regulating the transcription of the HIV-1 genome (4). Moreover, Tat also regulates transcription of other cellular promoters of phosphatases like PTEN and PP2A. These play crucial regulatory roles in apoptosis of HIV-1-infected CD4^+^ T cells (5).

Acetylation of specific lysine residues within the N-termini of selective core histones by HATs neutralizes positive charges on these amino acids, thereby weakening histone-DNA interactions and making the DNA more “open” or accessible to the transcriptional machinery [65, 66]. In contrast, recruitment of HDACs results in transcriptional repression. For example, recruitment of histone deacetylase-1 (HDAC1) by NF-*κ*B p50 was found to constitutively maintain nuc-0 and nuc-1 in a deacetylated state, thus keeping the chromatin in a condensed state, impairing RNA pol II recruitment and transcriptional initiation (Figure 3) [67]. The importance of HDAC1 has been shown by studies that have proposed a dynamic model for LTR regulation in T cells by two cellular transcriptional regulators YY1 and LSF [68]. They form a trimeric complex with HDAC1 at a region-spanning nucleotides from −10 to +27 of the HIV-1 LTR. Whereas LSF-1 binds to DNA, YY1 serves as an intermolecular bridge to anchor HDAC1 to this region [68].

Histone methylation can also have a number of effects on transcription. For example, methylation of lysine-9 (K9) on histone-3 (H3) by HMTs has been shown to be linked with transcriptional silencing, just as methylation of K4 that is associated with activation [69]. Accordingly, maintenance of the heterochromatic state at the integrated HIV-1 promoter has been shown to be mediated by the methyltransferase Suv39H1, which specifically mediates H3-K9 trimethylation, and the heterochromatin protein-1*γ*, which has been shown to recruit HMT [70–72]. Apart from methylation of histone, hypermethylation of CpG sites found within the HIV-1 LTR has also been shown to repress basal and activation-induced promoter activity, thereby inducing a state of latency [73].

Activation of HIV-1 transcription is mediated by host cell transcription factors and viral proteins via interactions with the cis-regulating elements in the LTR, along with protein-protein interactions in the regulatory pathway(s) [3]. LTR-basal transcription is driven primarily through cellular transcription factors such as Sp1 and NF-*κ*B, which help recruit the RNA pol II complex to the transcriptional start site. This process can be enhanced during cell activation stimulated by a number of cytokines [74]. The availability of host cell transcription factors and viral proteins regulates HIV-1 gene expression in the context of specific cell types, cell-cycle regulation, cellular differentiation, and cellular activation [75]. Sequestration of two critical transcription factors, NF-*κ*B and NFAT, in the cytoplasm of resting CD4^+^ T cells contributes to the repressive state of the HIV-1 LTR in these cells [76]. The paucity of these factors within the nucleus is reversed in response to activation signals. T cell receptor (TCR) cross-linking, cytokine stimulation (e.g., TNF-*α*, IL-7), or mitogens (e.g., protein kinase C activators like the phorbol ester PMA and prostratin) lead to nuclear translocation of these molecules and subsequent binding to overlapping cognate sites in the HIV-1 LTR, thereby upregulating basal as well as Tat-mediated promoter activity (Figure 3). TCR ligation also induces transcription and heterodimerization of the c-jun/c-fos complex AP-1, which is absent in resting T cells [77] and synergizes with NFAT and NF-*κ*B to promote HIV-1 gene expression. Basal and stimulated transcription produces predominantly short RNA as a result of the hypophosphorylated state of RNA pol II. However, an increasing number of longer transcripts encode for a pool of viral regulatory proteins, especially Tat, that eventually feedback to enhance the next stage of viral transcription, designated Tat-mediated transcription.

## 4. Tat-Mediated Transactivation of the HIV-1 ****LTR through Cyclin-Dependent Kinase 9 and**** Cyclin T1

HIV-1 transcription involves an early, Tat-independent phase and a late, Tat-dependent phase, and transactivation of the viral genome is a critical step in the viral replication cycle [3, 78]. In the presence of Tat, LTR-mediated transcriptional activity can be enhanced tens or hundreds of fold [79–82], whereas viral replication falls to nearly undetectable levels in the absence of Tat, and short transcripts (30–50 nucleotides) predominate [83–85]. Tat is a unique transcription factor in that it binds to the “UCU” bulge of the TAR, a cis-acting RNA enhancer element within the 5′ end of all viral transcripts. The TAR is located immediately downstream of the transcriptional start site in the HIV-1 LTR, encompassing nucleotides from +1 to +59 [86, 87], and is required for the function of the viral transactivator protein Tat. The Tat-TAR interaction acts to tether Tat and allow its interaction with the basal transcriptional machinery, thus increasing viral transcription and elongation [88, 89]. In a mature transcript, TAR adopts a hairpin structure including a six-nucleotide loop, a trinucleotide pyrimidine bulge, and an extensive duplex structure [86]. U23, in the bulge, is critical for Tat binding [2, 13, 90]; the other two neighboring residues C24 and U25 can be replaced by other nucleotides without affecting Tat binding. Another two regions above the bulge (G26-C39 and A27-U38) and one region below (A22-U40) also contribute to Tat binding [2, 13, 90]. Although the loop structure does not appear to be required for Tat binding, the residues in the loop have been shown to be required for Tat transactivation activity [90].

Specifically, HIV-1 Tat has been shown to associate with the P-TEFb, which is composed of cyclin T1 and cyclin-dependent kinase 9 (CDK9) [24, 91, 92]. This association occurs in a sequential manner. Once bound, CDK9 has been shown to phosphorylate the carboxy-terminal domain (CTD) of RNA pol II and promote transcription elongation [24, 91, 93]. Therefore, the lack of HIV-1 gene expression in latently infected cells might not only arise due to the absence of Tat but also as a result of extremely low levels of CDK9 and cyclin T1, as observed in resting CD4^+^ T cells [94]. In addition, mutations in the *tat* gene, the Tat-responsive element itself, might also contribute to the latent phenotype, as is evident from experiments performed in the U1 [95] and ACH-2 [96] cell lines, respectively. The cyclin T1 subunit of P-TEFb has been shown to interact with the activation domain of Tat and to bind to the central loop (+30 to +35) of TAR [92]. Once cyclin T1 binds to Tat, the CycT1-Tat complex has been shown to bind both the bulge and the loop regions of TAR with a higher affinity than Tat alone and to subsequently form the CycT1-Tat-TAR ternary complex [92, 97, 98].

With respect to sequence variation within the TAR region, different subtypes have been shown to have distinct TAR motifs, with most of the sequence variation occurring in the stem region [99]. Subtypes A/E and A contain a nucleotide deletion (T25) in the TAR bulge region (T23C24T25) leading to the formation of a two-nucleotide bulge [100–104]. This sequence alteration does not affect subtype B Tat binding to the subtype E TAR region. However, the studies concerning matched subtype A, A/E, or E TAR and Tat have not been reported [105]. A C-to-T change at position 24 in the bulge has been identified in subtypes C, D, F, most of G bulges, and 50% of subtype A bulges examined [104]. Additionally, a T-to-C change at position 2 of the loop structure has been consistently observed in subtype C [106]. Additional functional studies need to be performed to determine the impact of structural differences that may be important in the Tat-TAR-cyclin T1 interactions. Recent investigations have expanded the understanding of Tat-TAR-cycT1 interactions and have implicated the role of Tat acetylation in modulation of transcriptional elongation [107]. Moreover, mutation analysis of the N terminal of CycT1 [108] that impairs the transcriptional activity via compromising Tat-TAR-cycT1 interactions has laid the foundation for utilizing this aspect of HIV-1 transcriptional activation in novel therapeutic intervention strategies. These studies also add new aspects that extend our understanding of HIV-1-latency; however, adaptability of HIV-1 has hampered the use of these studies to any productive end. Another major issue is that most studies tend to examine these functional aspects of HIV-1 transcription in a subtype-specific manner and studies that encompass these variables are limited. Analyzing large databases of sequence information is still a tedious process, and development of more organized tools will encourage researchers to make better progress in this area.

## 5. Interaction of Tat with Other Proteins**** Involved in Transcription

The hypophosphorylated form of RNA pol II leads to the production of short RNA molecules (30–50 nucleotides in length including the entire length of the TAR sequence) as a result of premature termination of transcription. However, phosphorylation of the CTD of RNA pol II has been shown to prevent premature termination and promote the efficient elongation and production of full-length HIV-1 RNA transcripts [109]. Phosphorylation of the CTD of RNA pol II has also been shown to be important for the clearance of mediators from RNA pol II [110] (Figure 3). Transcription factor II H (TFIIH) is a part of the preinitiation complex involved in transcription, and a number of studies have shown that there are combinatorial networks of transcription factors and cofactors, such as P-TEFb, utilized by Tat to activate and repress gene expression [107, 111]. Tat and P-TEFb are recruited to viral preinitiation complexes prior to RNA transcription and are subsequently transferred to nascent RNA after initiation. In addition to regulating HIV-1 gene expression, Tat is known to be involved in dysregulating cellular function and altering cellular gene expression profiles; however, the mechanism by which Tat affects infected cells continues to be explored.

Tat also functions as a coactivator to recruit histone acetyltransferases, including CBP/p300 and PCAF to the LTR [112]. Tat-recruited HATs presumably acetylate histones in LTR-proximal nucleosomes, remodeling nuc-1, and potentiating transcription [60, 113]. In addition, Tat itself has been shown to be a substrate for the HAT enzyme activity associated with CBP/p300 and PCAF [114–118]. The HAT activity of CBP/p300 can also influence the activity of the NF-*κ*B p50 subunit. The increased acetylation of p50 leads to an increase in p50 DNA binding and the concomitant transcriptional activation of the HIV-1 promoter [119].

The SWI/SNF complex is another family of proteins that interacts with Tat and plays a role in the regulation of the HIV-1 promoter. The SWI/SNF proteins are integral components of the RNA pol II holoenzyme [120] and have the ability to disrupt DNA-histone contacts and allow access to transcriptional activators [120, 121]. In the context of HIV-1 transcription, SWI/SNF proteins are required for the transactivation ability of Tat and generation of mature full-length transcripts. Physical interaction between Tat and the remodeling subunits INI1 and Brm has also been observed [122, 123]. T cell activation by mitogenic stimuli induces recruitment of SWI/SNF complex subunits Jun-3, BRG-1, and ATF-3 [124]. In particular, BRG-1 has been shown to be recruited to the Ap1-III site located at the 3′ boundary of nuc-1 [124]. T cell activation also enhances the endogenous pools of inositol phosphate, increasing the activity of SWI/SNF by a yet-unexplained mechanism [125–127].

Tat has also been shown to facilitate enhanced transcriptional initiation through protein-protein interactions with Sp1 [128] (Figure 3). Tat residues 30–55 contact Sp1 and impact DNA-PK-mediated phosphorylation of Sp1, which increases gene expression driven by the HIV-1 LTR (Figure 3) [129]. Amino acids 56–101 of Tat contain the DNA-PK-binding domain. TFIIH has been shown to be able to bind the transactivation domain of Tat and phosphorylate the CTD of RNA pol II although a number of investigative groups have shown that different subunits of TFIIH may mediate Tat binding [130]. Interestingly, the ability of TFIIH to phosphorylate RNA pol II has been shown to be significantly increased after it was bound to Tat; in turn, the transactivation ability of Tat was enhanced in the presence of TFIIH [84]. Tat has been shown to be specifically associated with Tat-associated kinase, which corresponds to the *Drosophila* P-TEFb, composed of cyclin T1 and CDK9. Cyclin T1 interacts directly with the activation domain of Tat and has been shown to mediate high affinity and specific binding of Tat to TAR. After Tat binding to cyclin T1, CDK9 is recruited, and then CDK9 phosphorylates CTD of RNA pol II and promotes transcription elongation. Tat is able to effectively antagonize HIV-1 latency and promote active transcription by liberating P-TEFb from an inactive 7SK RNP complex. Moreover, Tat is proposed to engage in formation of a “super elongation complex” with elongation factors like ELL2, ENL, AFF4, PAF1, and others as previously reviewed [131] (Figure 3). Meanwhile, efficient transcription elongation of the HIV-1 genome in response to Tat has been shown to lead to more Tat synthesis and generate a Tat-dependent positive feedback loop. However, mouse cyclin T1 has a cysteine-to-tyrosine substitution at position 261 that may lead to weak binding of mouse cyclin T1 to Tat, resulting in Tat losing the ability to interact with TAR, which results in low transactivation levels. However, changing the tyrosine residue back to cysteine at position 261 renders mouse cyclin T1 fully functional in Tat transactivation, demonstrating the importance of this residue in the transactivation process [132].

It has been reported that Tat is also able to interact physically with C/EBP*βin vitro* and *in vivo* [133]. Tat amino acid residues from 47 to 67 are critical for interaction with C/EBP*β*, and specifically Tat increases the distribution of nuclear levels of C/EBP*β*. Moreover, Tat can activate C/EBP*β* in human U-373MG astroglial cells in a dose-dependent manner [134]. Recently, coexpression of Tat and C/EBP*β* has been shown to enhance C/EBP*β* binding to the HIV-1 LTR [135]. The N terminus of HIV-1 Tat (residues 1–26) has also been shown to bind to the transactivation domain (amino acids 1–96) within NFAT1 [136]. HIV-1 Tat enhances NFAT1-driven transcription in Jurkat T cells through a direct protein-protein interaction between the two proteins. 

Tat is a robust transactivating protein that induces a number of effects by modulating the expression of many cellular and viral genes. Tat was recently shown to be associated with the promoters of PTEN and PP2A subunits, and these interactions result in activation of apoptotic pathways in HIV-1-infected CD4^+^ T cells [137]. These studies reinforce the fact that Tat, besides regulating the HIV-1 promoter, also affects cellular promoters. In addition to all of these interactions, Tat also interacts with a wide array of proteins, which have been summarized in Table 1. As shown here, there are a number of proteins with which Tat has been shown to interact. However, very little is known concerning a majority of these interactions and how they may or may not contribute to HIV-1 pathogenesis. This may be one reason why Tat inhibitors, discussed below, have universally failed to date to be effective in the therapeutic arena. In fact, given the large numbers of proteins already identified, future studies will undoubtedly need to take into account the interactome that Tat has with respect to the various proteins involved to truly begin to design inhibitors to attack these interactions. However, modeling this concept will be a future area that will need addressing.

## 6. Molecular Diversity in Tat 

The high level of HIV sequence diversity generated during the course of HIV disease is, for the most part, due to the error-prone nature and low fidelity of reverse transcriptase, poor proofreading by the polymerase, and selective pressures exerted by the host immune response, combination antiretroviral chemotherapy, and perhaps other physiological pressures [3, 102, 138]. The HIV-1 genotypic variants and resultant phenotypes occur as important variables of viral replication during the course of the disease [139]. It has been reported that Tat can tolerate 38% sequence variation without any change in its transactivation potential [140]. 

Both blood-derived and brain-derived HIV-1 viruses show immense molecular heterogeneity between patients and HIV-1 subtypes [53]. The LTR and several HIV-1 genes including [tat], [env] (gp120 and gp41), [nef], and [vpr] have been linked to the pathogenesis of HIV-related neurologic disease [141]. The molecular diversity of HIV-1 Tat protein isolated from brains of patients infected with different HIV-1 subtypes has been examined. Recent studies examining Tat proteins representative of HIV-1 subtype B, C, and BF recombinants have demonstrated important structural and functional differences [142, 143]. BF recombinant HIV-1 isolates from Argentina appear to have a replicative advantage over subtype B isolates, possibly due to the differential ability of Tat to interact with the LTR. Subtype C Tat has been shown to be more highly ordered than subtype B Tat. In addition, subtype C Tat protein has been demonstrated to be consistently inferior to subtype B Tat in biological assays with respect to its ability to promote viral proliferation, induce TNF-*α* and IL-6 expression, and upregulate chemokine coreceptor expression [142]. However, studies have also shown that HIV-1 subtype C Tat exhibits greater transcriptional activity in the Jurkat CD4^+^ T cell line compared with subtypes B and E and that this higher level of transactivation is not LTR sequence dependent but rather because of variations in the C-Tat sequence at amino acid residues 57 (Arg in B and E, and Ser in C) and 63 (Glu in B, E, and C), which are within and close to the basic domain, respectively [144]. In addition, in HIV-1 subtype C Tat, a serine residue replaces a cysteine at position 31. This variation affects the biological function of Tat, resulting in a deficient chemoattractant activity, low ability to bind to chemokine receptor 2, and reduced ability to stimulate TNF-*α* production [142, 145, 146] without affecting Tat transactivation. More recently, signature pattern analysis identified five amino acid positions in Tat (21A, 24N, 29K, 40K, and 60Q) that contained signature residues unique for Indian HIV-1C [147]. Interestingly in the eight patients analyzed to date in the DrexelMed HIV/AIDS Genetic Analysis Cohort [148], which contains mostly subtype B-infected patients, all of the Tat sequences analyzed contain these important cysteines (data not shown). Some length variation in exon 2 and the absence of a critical cysteine in the cysteine-rich domain have also been found in subtype C Tat [106]. When one compares the structural and functional differences between subtypes B and C Tat proteins [142], subtype C Tat may have a relatively higher ordered structure and be less flexible than subtype B Tat. Analysis of subtype D Tat sequences revealed an in-frame stop codon in exon 2, which results in removal of the 13–16 amino acids from the C terminus of Tat [103, 149, 150]. Thus, emerging data encompassing the structure and function of the Tat protein across different subtypes have enabled us to better understand Tat-mediated effects. However, additional studies need to be performed to truly delineate the complexity of Tat. To accomplish this more sequencing data for all subtypes will have to be obtained to allow an even deeper understanding of how and why genetic variation evolves and what are the driving forces in this evolution especially on genes like *tat* that are not directly affected by antiretroviral therapy and therefore have direct selective pressures. The power of having large data sets can be demonstrated in a recent study which has identified two residues in Tat, positions 35 and 39, which appear to be coevolved. These residues, however, have two distinct functions with respect to the transactivation of the HIV-1 LTR-binding P-TEFb and promoting P-TEFb phosphorylation of the C-terminal domain in RNAPII, respectively [151]. In addition, understanding the genetic diversity of Tat in multiple subtypes has become and will continue to be increasingly important as vaccines in development will need to account for immunity to all of these variations.

## 7. Tat Genetic Variation and HIV-1-Associated ****Neurological Disorders 

In addition to transactivation of the viral LTR, Tat exhibits a range of biological properties relative to HIV-1 pathogenesis [152], including the intracellular regulation of host gene expression to facilitate viral production as well as the extracellular detrimental effects on the cells of the immune and nervous systems. HIV-1 induces pathological consequences in a number of end organs including the brain [7, 153]. More than 30% of AIDS patients suffer from some form of HIV-1-induced neurological impairment including HIV-1-associated dementia (HAD) as well as other more subtle minor neurocognitive disorders [154, 155]. Despite the widespread use of highly active antiretroviral therapy and the resultant decrease in the incidence of HAD, the prevalence of HAD and other milder forms of HIV-related neurological disease has become increasingly common problems with respect to the clinical management of HIV/AIDS [156]. In particular, Tat has been implicated in the pathogenesis of HIV-associated neurological disease including HAD via a variety of mechanisms [157]. The neurotoxicity of Tat is further supported by the observation that the mRNA levels for Tat are elevated in brain extracts of patients with HAD [158]. Tat has been shown to act as an intracellular and extracellular mediator of neurotoxicity and to play a critical role in contributing to neurological injury in HAD [159]. Tat protein is secreted by HIV-1-infected cells and acts by diffusing through the cell membrane. It appears to act as a secreted, soluble neurotoxin and induces HIV-1-infected macrophages and microglia to release neurotoxic substances [160–162]. Some Tat variants have been reported to be dysfunctional with respect to LTR transactivation and may contribute to viral latency under certain conditions while still being able to stimulate the transcription of a number of cytokine genes [95]. Tat can also cooperate with cellular factors to enhance the neurotoxic effects on host cells [163]. Tat and cytokines IFN-*γ* and TNF-*α* have also been demonstrated to synergistically increase expression of CXCL10 in human astrocytes, which provide an important reservoir for the generation of inflammatory mediators, for instance, CXCL10 as a neurotoxin and a chemoattractant [164]. 

Phylogenetic analyses of Tat sequences from patients with and without HAD have shown clustering of sequences with respect to clinical diagnosis of neurological impairment as well as tissue of origin [165, 166]. Comparisons of matched brain and spleen-derived Tat sequences have suggested that greater sequence homology exists among brain-derived Tat clones than that observed between brain and spleen-derived clones [166]. Another study also showed sequence variations within patients segregated as CNS and non-CNS *tat* genes [167]. Additionally, significant sequence heterogeneity exists within brain-derived Tat in domains associated with viral replication and intracellular transport [166]. Nonsynonymous versus synonymous mutation rates among brain-derived Tat sequences isolated from patients with neurocognitive impairment were shown to be significantly greater than those isolated from patients without clinical evidence of neurological disease [165]. Importantly, most of the mutations present in the HAD-associated Tat sequences were located in the augmenting region (residues 57–78 amino acids), which affects viral replication. Interestingly, in these studies, variations at position 74 and 100 were correlated to Tat sequences isolated from brain-derived sequences. Collectively, results from these studies suggest that differing selective pressures act on individual HIV-1 genes within the CNS and that this differential selective pressure may influence both the development and subsequent severity of neurocognitive impairment.

Participants in a current longitudinal study of patients in the DrexelMed HIV/AIDS Genetic Analysis Cohort have had their LTR and their *env* and *tat* genes and proteins, where appropriate, analyzed from peripehral blood. The LTR was analyzed as a result of previous studies that identified the potential for single nucleotide polymorphisms in the LTR to be predictive of neurocognitive impairment [168–170]. Patients within the DrexelMed HIV/AIDS Genetic Analysis Cohort are followed longitudinally, with scheduled visits every 6 months. At each visit, patients are interviewed for clinical and social history, a blood sample is collected, and neurocognitive status is evaluated with a minineurological exam [171]. Recently, eight patients were analyzed with respect to Tat genetic variation. Longitudinal analyses of these 8 patients showed 7 of the patients had exhibited NI at some point in their clinical history; of these 7 patients, 3 patients had current NI and all 3 had Tat containing a change at amino acid position 100 (amino acid 100). Of the remaining 4 patients, 2 had the change at amino acid 100 with their following visit showing NI; the final 2 did not have a change at amino acid 100; however, these patients were lost to follow up for 23–36 months, at which time they presented with NI. The one patient without NI did not have a change at amino acid 100. Given the previous studies, which have identified this amino acid position as indicative of brain-derived sequences, this observation may point to the fact that a change at this amino acid position may occur in the periphery in patients before the onset of NI (perhaps in the HIV-1-infected monocyte-macrophage compartment). 

The genetic variation observed within Tat has also been shown to alter the function of this protein and relate to pathogenesis. Importantly, HIV-1 Tat derived from HAD patients has been associated with greater neuronal death both *in vitro* and *in vivo* compared with Tat from non-HAD patients. This characteristic has been attributed, in part, to enhanced MMP-2 expression induced by brain-derived HIV-1 Tat variants [172]. Interestingly, however, these same brain-derived Tat isolates also appear to be limited in their ability to enhance viral gene expression despite the increased activation of host transcriptional machinery [173]. This observation differs from other reported studies, which showed that a subset of patients demonstrated reduced transactivation capacity of CNS-derived Tat proteins compared to those from matched lymphoid tissues; however, overall Tat proteins from the CNS, when compared to lymphoid compartments, maintained similar levels of transactivation function [167]. However, one must remember that these viral gene activation studies were all performed with a viral regulatory region derived from a non-CNS tissue source and may therefore not be naturally compatible with respect to optimal LTR activation by a Tat protein selected for CNS replication. This observation is of particular importance because previous studies [168, 174, 175] have demonstrated that LTRs derived from the CNS are likely to be structurally and functionally different from LTRs derived from other tissue sources. In fact, a current study has shown that Tat transactivates the corresponding HIV-1-infected patient-derived colinear LTR better than a non-co-linear Tat protein. These Tat clones were shown to have nonconsensus variations compared with IIIB or the consensus B sequence of Tat that might contribute to the alteration in their function [171]. Taken together, these reports point to the notion that genetic diversity of HIV-1 Tat likely contributes to the establishment and severity of HIV-1-associated neurological disease. However, additional studies of this nature are warranted. For instance, are there certain Tat residues that induce increased Tat secretion from cells both in the periphery as well as in the CNS? Are there variations that induce more neurotoxic effects? Are the Tat variants isolated from vRNA versus integrated DNA similar or different? 

## 8. HIV-1 Tat as a Therapeutic Target

The HIV-1 Tat protein has long remained an attractive target for therapeutic intervention owing to its essential role in viral gene expression and activation of the HIV-1 LTR. As discussed before, Tat and the P-TEFb complex bind to TAR to promote efficient transcription of the full-length HIV genome. The expanding knowledge of Tat functional properties and its interactions with other cellular and viral partners has led to the identification of a varied range of compounds that can inhibit different Tat functions. The Tat and HIV-1 transactivation inhibitors fall broadly into the following categories: (1) inhibitors targeting TAR RNA (2), inhibitors targeting Tat protein, and (3) Tat-P-TEFb interaction inhibitors. In this section, we review the current status of the development of therapeutic strategies that target Tat and its functional interactions in the process of HIV-1 transcription (Table 2). 

Compounds against the TAR RNA are the most numerous because they would block the primary functional interaction of Tat in the process of HIV-1 transcription. In this category, the compounds can be broadly divided into three classes: (1) peptide based, (2) oligonucleotide based, and (3) small-molecule based (for more detailed information refer to a previously published review [176]). It is well established that the arginine-rich motif of the Tat protein is required for it to bind to the TAR RNA trinucleotide bulge region [15, 90, 177]. Peptides corresponding to this region were found to compete for Tat binding and were shown to inhibit HIV-1 replication [32, 178]. Compounds directly binding to the three-base bulge of TAR RNA include 6-aminoquinolone [179, 180], quinoxaline-2, 3-diones [181], pyridine oxide derivatives such as JPL-32 [182, 183], and acridine derivatives such as CGP64222 and CGP40336A [33, 34]. All of these compounds exhibit strong inhibition of the Tat-TAR interaction by binding to the three-base bulge of TAR RNA. The peptide CGP64222 was later shown also to interact with CXCR4 [184], the chemokine receptor that acts as a coreceptor for the X4 or dual tropic HIV-1 strains. Other drugs that specifically interact with the bulge in TAR RNA include biscationic diphenylfuran derivatives and a new class of polyamine-acridine-based compounds [34, 185]. Aminoglycoside antibiotics such as neomycin and streptomycin and neamine and its derivatives have been shown to specifically bind RNA molecules and to block the conformation of the Tat-TAR complex by targeting the structure of TAR RNA [181, 186, 187]. Besides the aforementioned Tat-TAR inhibition strategies, the developments in the field of RNA interference have yet to be applied in full force for achieving a more targeted inhibition [188, 189]. 

Compounds binding directly to the Tat protein could inhibit HIV-1 replication. To achieve this goal, targeting the basic domain of Tat would be relevant because this very domain is required for nuclear localization, transactivation through TAR binding, and also for extracellular release and cellular uptake [190, 191]. It has been shown that, in the extracellular compartment, the basic domain of Tat can be targeted by several polyanions [192] such as heparin and heparan sulfates [40], thrombospondin [193], polysulfonated distamycin A derivatives [194], and sulfated polysaccharides [195], thereby blocking its internalization and also its extracellular activities. Another compound targeting Tat is the negatively charged polyacrylic acid, which could inhibit the Tat-TAR interaction with high affinity to Tat peptide, thereby blocking HIV-1 replication [196]. Negatively charged small molecules, such as the stilbene derivative, CGA-137053, was shown to inhibit HIV-1 replication by directly binding to Tat and inhibiting the formation of the Tat-TAR complex at low nanomolar concentrations [39]. One important aspect, which must be considered, is that most of these interactions are largely due to electrostatic interactions with limited sequence specificity. Therefore, it would be highly relevant to evaluate their specificity in a relevant model system before they can be exploited in any therapeutic intervention strategy. Some transdominant-negative Tat mutants have also been shown to be potential antiviral therapeutics because they could inhibit the transactivation function of Tat, thereby inducing latency during viral infection [197–200]. Moreover, various biopolymeric drugs and anti-Tat antibodies have been demonstrated to be effective in inhibiting the extracellular activity and cellular uptake of Tat protein [176, 201]. Thus, targeting specific, conserved conformational epitopes on Tat might prove to be more beneficial. This approach can be aided tremendously by the emerging structural data on Tat. Some insights have already been provided by *ab initio* molecular dynamic studies on the Tat NMR structure and structural conformations of TAR [202, 203]. It would also be relevant to evaluate combination drug formulations to achieve an inhibition of the functional interactions of Tat at several levels [204]. This combination approach may also facilitate strategies that use relatively lower concentrations of these compounds that might improve overall toxicity levels. It might also be useful in targeting the small pool of latently infected cells that may hinder clearing the virus from the system.

The third approach is to use the understanding of the Tat-P-TEFb interaction to develop interventions to disrupt this interaction or to reduce the stability of this complex. P-TEFb (CDK9/cyclin T1) is an essential cofactor for Tat-mediated transactivation, and selective inhibition blocks HIV-1 replication without affecting cellular transcription, thus making it a potential target for anti-HIV-1 therapy. To this end, P-TEFb inhibitors such as 5,6-dichlororibofuranosylbenzimidazole, a purine nucleoside analogue; flavopiridol, a small molecular cyclin-dependent kinase inhibitor [23, 24]; Seliciclib, an inhibitior of CDK2/cyclin E and/or P-TEFb [25] have been evaluated in various *in vitro* studies and have been shown to effectively reduce HIV-1 replication. However, long-term HIV-1 replication studies showed that these inhibitors were more cytotoxic and less efficacious against HIV-1 in the primary cell cultures [205]. The failure of these known kinase inhibitors in providing anti-HIV efficacy has prompted studies to revisit the Tat-P-TEFb complex for small molecule inhibitors. In this study, molecular dynamics simulations are being utilized to understand the nature of interactions of Tat with CDK9 and Cyclin-T1 in a dynamic mode. These interactions are further mapped on to a pharmacophore-based screening paradigm to design small molecule inhibitors that show potent HIV-1 efficacy and low toxicity (Kortagere and Wigdahl, unpublished results).

In alternative approaches, promising results were demonstrated using anti-CycT1 human single-chain antibodies that targeted the cyclin domain and the TAR recognition motif, using transiently transfected cell lines [132] and stably transfected cells [206]. Protein chimeras like fusion of a truncated human CycT1 and a mutant CDK9 protein that lacked autophosphorylation activity have demonstrated the inhibition of Tat-mediated transactivation and HIV-1 gene expression [207]. Intracellular proteins that inhibit P-TEFb-like HEXIM1 have been evaluated to suppress HIV-1 replication [208, 209]. Moreover, the inhibition of a transcriptional coactivator like PCAF has been evaluated using antibodies against the bromodomain of PCAF [210]. These studies establish a therapeutic rationale, but more specificity is desired because targeting proteins involved in cellular homeostasis and activation pathways may have detrimental effects on the cells. The same complexity applies to studies that propose targeting specific posttranslation modifications on Tat such as phosphorylation [211] and acetylation [107]. Moreover, most of these studies are done in irrelevant cell types that dilute their therapeutic promise. Another aspect that gets overlooked is that it is extremely difficult to evaluate this effect since any such intervention will have an impact on normal cellular pathways. This underscores the value of in-depth analysis of the functional interactions in the HIV-1 replication paradigm because it would yield more specific targets with minimal host toxicity. Another aspect that has not been investigated in detail is the genetic variations observed within the Tat-coding sequences and how they might impact the structure and function of this vital transactivator. Efforts in our laboratory and others have indicated variation in the transactivation potential of different Tat sequences from HIV-1-infected patients (unpublished observations). Moreover, it has been established that sequence variation within specific domains of Tat was associated with increased viral replication and TNF-*α* production [141, 166, 212, 213]. These observations along with results that have shown that some Tat sequences exhibit minimal transactivation potential but have an ability to activate host gene expression [173] provide new directions where this aspect of Tat sequence variability can be included in strategies directed against Tat. The ideal outcome would be to achieve a competitive inhibition by using defective Tat mutants to inhibit Tat function, but a thorough understanding selected aspects of Tat function is a prerequisite to this line of investigation. These efforts again will be aided immensely by incorporating testing that includes Tat sequence and functional information across subtypes. 

## 9. Conclusions

More than two decades of investigations have established the central role of Tat in the activation of HIV-1 LTR. Genetic- and structure/function-based studies have enabled us to understand the functional intricacies of Tat-dependent functions. All of these studies have motivated a number of researchers to use Tat as an important target in combination antiretroviral therapies, but to date none of these have materialized into clinically effective antiviral agents. One crippling factor has been the inability to assess the functions of Tat in relevant systems at a concentration that would be closer to that encountered *in vivo*. Concentrating efforts in a direction to elucidate the protein-protein interactome established by Tat will go a long way toward targeting specific breakpoints in HIV-1 pathogenesis. Isolation of mutant sequences of Tat from sites like the brain can also be used to identify tissue-specific functions of Tat that may have great bearing on long-term use of Tat inhibitors. Moreover, it would be an important effort to consolidate studies concerning the structural information and functional interactions of Tat across different HIV subtypes and use this information to increase the spectrum of subtypes susceptible to Tat-based therapeutic inhibitors. Eradicating latent reservoirs by the elimination of integrated HIV-1 provirus or irreversibly blocking LTR activation or Tat transactivation activity will provide the next major step forward in controlling the HIV-1 pandemic. 

## Figures and Tables

**Table 1:** List of all known Tat protein interactions and the nature of the functional activity compiled from PubMed (http://www.ncbi.nlm.nih.gov/pubmed/) and HIV-1, Human Protein Interaction Database (http://www.ncbi.nlm.nih.gov/RefSeq/HIVInteractions/).

Human protein	Type of activity with Tat
2^′^-5^′^-Oligoadenylate synthetase 2 isoform 1	Interacts
2^′^-5^′^-Oligoadenylate synthetase 3	Interacts
2^′^,3^′^-Cyclic nucleotide 3^′^ phosphodiesterase	Activates
2^′^,5^′^-Oligoadenylate synthetase 1 isoform 2	Interacts
8-Oxoguanine DNA glycosylase isoform 1a	Upregulates
Actin, gamma 1 propeptide	Induces rearrangement of
Activated RNA polymerase II transcription cofactor 4	Binds
Adaptor-related protein complex 2, alpha 1, 2 beta-1, mu-1, and sigma-1	Interacts
Adenosine A2a receptor	Inhibited by
Adenylate cyclase 2–9	Inhibits
AFF4	Involved in transcription
Albumin precursor	Induces release
Alpha 1 actin precursor	Induces rearrangement
Alpha 1 type-I collagen preproprotein	Inhibits
Alpha 2 actin	Induces rearrangement
Alpha 2 type-I collagen	Inhibits
Alpha isoform of regulatory subunit A, B55, protein phosphatase 2	Modulated by
Alpha-2-macroglobulin precursor	Inhibits
AMP-activated protein kinase gamma 2 subunit isoform a	Activates
AMP-activated protein kinase, noncatalytic gamma-1 subunit isoform 1	Activates
Amyloid beta A4 protein precursor, isoform a	Inhibits
Annexin A2 isoform 2	Downregulates
Apolipoprotein E precursor	Inhibited by
ATP-binding cassette subfamily B, C member 1	Upregulates
ATP-dependent DNA helicase II	Interacts
ATP-dependent DNA helicase II, 70 kDa subunit	Interacts
Autoantigen La	Interacts
B-cell CLL/lymphoma 11B isoform 1	Binds
B-cell lymphoma 6 protein	Upregulates
B-cell lymphoma protein 2 alpha isoform	Interacts
Baculoviral IAP repeat-containing protein 3	Upregulates
BCL2-antagonist of cell death protein	Induces phosphorylation
BCL2-associated athanogene isoform 1L	Upregulates
BCL2-associated X protein isoform beta	Interacts
BCL2-like 1 isoform 1	Upregulates
BCL2-like 11 isoform 6	Interacts
Beta actin	Induces rearrangement
Beta isoform of regulatory subunit A, B55 and B56, protein phosphatase 2 isoform a	Modulated by
Beta tubulin 1, class VI	Binds
Beta-2-microglobulin precursor	Downregulates
Bone-morphogenetic protein 1, 2 isoform 1, precursor	Upregulates
Bone-morphogenetic protein receptor type-II precursor	Downregulates
Brain adenylate cyclase 1	Inhibits
Breast cancer antiestrogen resistance 1	Induces phosphorylation
BTAF1 RNA polymerase II, B-TFIID transcription factor-associated, 170 kDa	Interacts with
c-Src tyrosine kinase	Activates
Calcium/calmodulin-dependent protein kinase I	Downregulates
Calcium/calmodulin-dependent protein kinase IIA, IIB isoform 1	Inhibits
cAMP-responsive element-binding protein 1 isoform A	Activates
cAMP-responsive element modulator isoform v	Activates
cAMP-dependent protein kinase catalytic subunit beta isoform 1	Activates
cAMP-dependent protein kinase, regulatory subunit alpha 1 and beta 2	Activates
cAMP-specific phosphodiesterase 4D	Activates
Cardiac muscle alpha actin 1 proprotein	Induces rearrangement
Caspase 3 preproprotein	Activates
Caspase 8 isoform A precursor	Upregulates
CC chemokine receptor 3	Binds
CCAAT/enhancer binding protein	Binds
CCAAT/enhancer binding protein beta	Binds
CD180 antigen	Downregulates
CD28 antigen	Interacts
CD3D antigen, delta polypeptide isoform A precursor	Interacts
CD3E antigen, epsilon polypeptide (TiT3 complex)	Interacts
CD3G gamma precursor	Interacts
CD4 antigen precursor	Upregulates
CD40 antigen isoform 1 precursor	Upregulates
Cell division cycle 2 protein isoform 1	Interacts
Cell division cycle 20 and 25C protein	Downregulates
Cell division cycle 37 protein	Regulated by
Cell division cycle 6 protein	Interacts
Chaperonin containing TCP1, subunit 4 (delta)	Interacts
Chemokine (C motif) ligand 1, 3, 7	Upregulates
Chemokine (C-C motif) receptor 1	Modulates
Chemokine (C-C motif) receptor 2 isoform B	Binds
Chemokine (C-C motif) receptor 4	Upregulated by
Chemokine (C-C motif) receptor 5	Upregulates
Chemokine (C-X-C motif) ligand 12 (stromal cell-derived factor 1) isoform beta	Interacts
Chemokine (C-X-C motif) receptor 3	Inhibits
Chemokine (C-X-C motif) receptor 4 isoform b	Binds
Chemokine (C-X3-C motif) ligand 1	Inhibited by
Chemokine C-C motif ligand 4 isoform 1 precursor	Upregulates
Chromobox homolog 5 (HP1 alpha homolog, Drosophila)	Inactivates
Class II transactivator	Inhibits
Claudin 1	Downregulates
Claudin 5	Downregulates
Cleavage and polyadenylation specific factor 3, 73 kDa	Upregulates
Cofactor of BRCA1	Associates with
Cofilin 1 (nonmuscle)	Downregulates
Cofilin 2 (muscle)	Downregulates
Collagen, type-III, alpha 1 preproprotein	Upregulates
Colony-stimulating factor 2 precursor	Upregulates
Complement component 1 inhibitor precursor	Upregulates
Complement component 1, q subcomponent-binding protein precursor	Binds
Core histone macro-H2A2.2	Binds
CREB-binding protein isoform a	Binds
CREB3: cAMP-responsive element binding protein 3 (luman)	Inhibited by
CTD (carboxy-terminal domain, RNA polymerase II, polypeptide A) phosphatase, subunit 1 isoform FCP1a	Binds
CXCL16	Interacts
Cyclin A, A1, E1	Interacts
Cyclin B1	Upregulates
Cyclin C isoform a	Interacts
Cyclin D1	Downregulates
Cyclin D3	Interacts
Cyclin H	Binds
Cyclin T1	Binds
Cyclin T2 isoform a	Binds
Cyclin-dependent kinase 2 isoform 1	Enhances
Cyclin-dependent kinase 4	Interacts
Cyclin-dependent kinase 5	Downregulates
Cyclin-dependent kinase 7	Binds
Cyclin-dependent kinase 8	Interacts
Cyclin-dependent kinase 9	Binds
Cyclin-dependent kinase inhibitor 1A	Activates
Cyclin-dependent kinase inhibitor 1B	Interacts
Cytochrome c	Induces release of
Cytoplasmic nuclear factor of activated T cells 3 isoform 1	Activates
Cytoplasmic nuclear factor of activated T cells 4	Activates
Delta isoform of regulatory subunit B56, protein phosphatase 2A isoform 1	Modulated by
Dicer1	Interacts
Dipeptidyl peptidase IV	Inhibits
Diubiquitin	Ubiquitinated by
DNA-dependent protein kinase	Modulates HIV gene expression
DNA-directed RNA polymerase II polypeptides	Interacts
DNA polymerase epsilon catalytic subunit	Interacts
DNA-damage-inducible transcript 3	Enhanced by
DNA-directed RNA polymerase III 39 kDa polypeptide F	Activates
Dopamine transporter	Inhibits
Downregulator of transcription 1	Inhibits
E1A-binding protein p300	Complexes with
E2F transcription factor 1	Inhibited by
E2F transcription factor 4	Recruits
Early growth response 1, 2, 3	Binds
Egr-1 in astrocytes	Tat-mediated toxicity in astrocytes
Elongation factor RNA polymerase II	Interacts
Elongin B isoform a	Interacts
Endonuclease G precursor	Activates
Endothelial differentiation-related factor 1 isoform alpha	Downregulates
Endothelin 1	Upregulates
Enolase 1	Inhibits
EP300	Binds
Epidermal growth factor (beta urogastrone)	Interacts
Epsilon isoform of regulatory subunit B56, protein phosphatase 2A	Modulated by
Eukaryotic translation elongation factor 1 alpha 1, 2	Interacts
Eukaryotic translation initiation factor 2-alpha kinase 2	Interacts
Excision repair cross-complementing rodent repair deficiency, complementation group 2 protein	Binds
Excision repair cross-complementing rodent repair deficiency, complementation group 3	Binds
Fas ligand	Interacts
Fc fragment of IgG, low affinity IIIa, receptor for (CD16)	Inhibits
Fibroblast growth factor 1 (acidic) isoform 1 precursor	Upregulates
Fibronectin 1 isoform 3 preproprotein	Competes with
Fms-related tyrosine kinase 1 (vascular endothelial growth factor/vascular permeability factor receptor)	Binds
Furin preproprotein	Cleaved by
Galectin 3	Upregulates
Gamma isoform of regulatory subunit B55, protein phosphatase 2 isoform a	Regulated by
Gastrointestinal glutathione peroxidase 2	Modulates
GATA-binding protein 2	Synergizes with
GCN5 general control of amino-acid synthesis 5-like 2	Binds
Gelsolin isoform a precursor	Downregulates
General transcription factor iib, e, f, and h	Interacts
General transcription factor IIIC, polypeptide 1, alpha 220 kDa	Activates
GLI-Kruppel family member GLI2	Synergizes with
Glial fibrillary acidic protein	Upregulates
Glucocerebrosidase precursor	Interacts
Glucose-6-phosphate dehydrogenase isoform a	Activates
Glutamate receptor, ionotropic, N-methyl-D-aspartate 3A, 3B	Activates
Glutamate receptor, metabotropic 1	Activates
Glutathione peroxidase 1 isoform 1, 4, 5, 6, 7	Downregulates
Glutathione synthetase	Modulates
Glycogen synthase kinase 3 beta	Activates
Glycophorin A precursor	Downregulates
Granulin precursor	Binds
Grb2	Binds through SH3 domains
Growth factor receptor-bound protein 2 isoform 1	Recruits
Growth hormone 1 isoform 1	Inhibited by
H2A histone family members	Binds
H2B histone family members	Binds
H3 histone family members	Binds
H4 histone family members	Binds
Heat shock 70 kDa proteins	Regulated by
Heparan sulfate proteoglycan 2	Interacts
Heparanase	Interacts
Hexamethylene bis-acetamide inducible 1, 2	Inhibited by
Histone 1, h2ad	Binds
Histone 2, H4	Binds
Histone deacetylase 1	Inhibited by
Histone h2a	Binds
Histone H2A	Binds
Histone H2B	Binds
Histone H4	Binds
HIV TAT specific factor 1	Stimulated by
HIV-1 Tat interactive protein 2, 30 kDa	Stimulates
HIV-1 Tat interactive protein, 60 kDa isoform 1	Binds
HMT1 hnRNP methyltransferase-like 6	Methylated by
Human immunodeficiency virus type-I enhancer binding protein 1	Enhanced by
Inhibitor of DNA-binding 1 isoform a	Upregulates
Ini1/hSNF5	Interacts
Inositol 1,4,5-triphosphate receptor, type 1, 2, 3	Interacts
Insulin-like growth factor 1 (somatomedin C)	Inhibited by
Insulin-like growth factor-binding protein 4 precursor	Binds
Integrin alpha 3 isoform a precursor	Activates
Integrin alpha 5 precursor	Binds
Integrin alpha L precursor	Inhibits
Integrin beta 1 isoform 1A precursor	Interacts
Integrin beta 4 isoform 1 precursor	Interacts
Integrin beta chain, beta 3 precursor	Interacts
Integrin, beta 2 precursor	Inhibits
Integrin, beta 5	Interacts
Intercellular adhesion molecule 1 precursor	Interacts
Interferon regulatory factor 1	Binds
Interferon regulatory factor 7 isoform a	Upregulates
Interferon-stimulated gene 20 kDa	Upregulates
Interferon-induced protein 35	Upregulates
Interferon-induced protein with tetratricopeptide repeats 3	Upregulates
Interferon-induced, hepatitis C-associated microtubular aggregate protein	Upregulates
Interferon, alpha 1	Interacts
Interferon, alpha-inducible protein (clone IFI-15 K)	Upregulates
Interferon, alpha-inducible protein 27	Upregulates
Interferon, beta 1, fibroblast	Interacts
Interferon, gamma	Inhibited by
Interferon, gamma-inducible protein 16	Upregulates
Interleukin 1 receptor antagonist isoform 2	Upregulates
Interleukin 1 receptor, type I precursor	Upregulates
Interleukin 1, alpha proprotein	Downregulates
Interleukin 1, beta proprotein	Downregulates
Interleukin 10 precursor	Inhibited by
Interleukin 12A precursor	Downregulates
Interleukin 12B precursor	Downregulates
Interleukin 13 precursor	Induces release of
Interleukin 16 isoform 1 precursor	Inhibited by
Interleukin 19 isoform 1 precursor	Upregulates
Interleukin 2 precursor	Downregulates
Interleukin 2 receptor beta precursor	Downregulates
Interleukin 2 receptor, alpha chain precursor	Upregulates
Interleukin 20 precursor	Upregulates
Interleukin 3 precursor	Upregulates
Interleukin 4 isoform 1 precursor	Interacts
Interleukin 6 (interferon, beta 2)	Activated by
Interleukin 6 receptor isoform 1 precursor	Upregulates
Interleukin 7 precursor	Inhibits
Interleukin 7 receptor precursor	Inhibits
Interleukin 8 precursor	Downregulates
Jun oncogene	Binds
Karyopherin beta 1	Binds
Kinase insert domain receptor (a type-III receptor tyrosine kinase)	Interacts with
Kinesin family member 2C	Downregulates
Kruppel-like factor 9	Enhanced by
Lactate dehydrogenase A	Downregulates
Lactate dehydrogenase B	Downregulates
Lamin A/C isoform 1 precursor	Binds
Lamin B1	Binds
Lamin B2	Binds
Laminin alpha 5	Upregulates
Laminin subunit beta 3 precursor	Upregulates
Laminin, alpha 1 precursor	Upregulates
Laminin, alpha 4 precursor	Upregulates
Laminin, beta 1 precursor	Upregulates
Laminin, beta 2 precursor	Upregulates
Laminin, beta 4	Upregulates
Laminin, gamma 1 precursor	Upregulates
Laminin, gamma 2 isoform a precursor	Upregulates
Laminin, gamma 3 precursor	Upregulates
Low affinity immunoglobulin gamma Fc region receptor III-B precursor	Inhibits
Low-density lipoprotein-related protein 1	Binds
LSD1/KDM1	Modulates transcription
Lymphocyte-specific protein tyrosine kinase precursor	Activates
Lymphotoxin alpha precursor	Upregulates
MAD, mothers against decapentaplegic homolog 6	Downregulates
Major histocompatibility complex, class I	Downregulates
Major histocompatibility complex, class II	Downregulates
Manganese superoxide dismutase isoform A precursor	Downregulates
Mannose receptor C type-1 precursor	Downregulates
Mannose receptor C type 2	Downregulates
Matrix metalloproteinase	Activates
Menage a trois 1 (CAK assembly factor)	Binds
Metallothioneins	Upregulates
Methyltransferase-like protein 1 isoform a	Downregulates
Microtubule-associated deacetylase HDAC6	Tat acetylation and transactivation
Mitogen-activated protein kinases	Activates
Mothers against decapentaplegic homolog 3	Inhibited by
Mothers against decapentaplegic homolog 4	Inhibited by
Mouse double minute 2 homolog isoform MDM2	Regulated by
Myc protooncogene protein	Upregulates
MyoD family inhibitor domain containing isoform p40	Binds
Myxovirus resistance protein 1	Upregulates
Nasal embryonic LHRH factor	Associates with
Nerve growth factor, beta polypeptide precursor	Inhibits
Neutrophil cytosolic factor 1	Interacts
Nitric oxide synthase 2A isoform 1	Inhibits
Nitric oxide synthase 3 (endothelial cell)	Upregulates
NMDA receptor 1 isoforms	Activates
Notch 2 preproprotein	Interacts
Nrf2 transcription factor	Fusion to Tat
Nuclear factor I/C isoform 1	Synergizes with
Nuclear factor kappa-B, subunit 1	Interacts
Nuclear factor of activated T cells, cytosolic component 1 isoform A	Activates
Nuclear factor of activated T cells, cytoplasmic, calcineurin-dependent 2 isoform B	Binds
Nuclear factor of kappa light polypeptide gene enhancer in B cells	Activates
Nuclear receptor coactivator 1 isoform 1	Stimulates
Nuclear receptor coactivator 2	Stimulates
Nuclear receptor coactivator 3 isoform a	Enhanced by
Nuclear receptor subfamily 2, group F, member 1	Interacts
Nuclease-sensitive element binding protein 1	Binds
Nucleophosmin 1 isoform 1	Binds
Nucleosome assembly protein 1	Interacts
P-TEFb	Binds
p21-activated kinase 1	Interacts
p300/CBP-associated factor	Interacts
p65	Binds
Paired mesoderm homeobox 1 isoform pmx-1a	Downregulates
Pancreas-enriched phospholipase C	Activates
Paxillin	Induces phosphorylation of
Phorbolin 1	Upregulates
Phosphatase and tensin homolog	Downregulates
Phosphodiesterases	Activates
Phosphoinositide-3-kinases	Interacts with
Phosphoinositide-specific phospholipase C beta 1 isoform a	Activates
Phospholipase C isoforms	Activates
Plasma glutathione peroxidase 3 precursor	Downregulates
Plasminogen activator inhibitor-1	Modulates
Platelet-activating factor acetylhydrolase, isoform Ib, alpha subunit (45kDa)	Interacts
Poly(ADP-ribose) polymerase family, member 1	Regulates
Poly(A) polymerase alpha	Regulates
Poly(A) polymerase beta (testis specific)	Regulates
Poly(A) polymerase gamma	Regulates
Polymerase (DNA directed), beta	Upregulates
Polymerase (RNA) III (DNA directed) polypeptides	Activates
Polypyrimidine tract-binding protein 2	Interacts
Polypyrimidine tract-binding protein 1 isoform a	Interacts
POU domain, class 2, transcription factor 1	Binds
PRF1: perforin 1 (pore forming protein)	Downregulates
Prion protein preproprotein	Upregulates
PRKC, apoptosis, WT1, regulator	Upregulates
Programmed cell death 11	Modulated by
Proliferating cell nuclear antigen	Interacts
Promyelocytic leukemia protein isoform 1	Regulated by
Prostaglandin-endoperoxide synthase 2 precursor	Upregulates
Proteasome (prosome, macropain) 26S subunit, non-ATPase, 6	Interacts
Proteasome (prosome, macropain) activator subunit 4	Interacts
Proteasome 26S ATPase subunits	Interacts
Proteasome 26S non-ATPase subunits	Interacts
Proteasome activator subunits	Interacts
Proteasome alpha subunits	Interacts
Proteasome beta subunits	Interacts
Proteasome inhibitor subunit 1 isoform 1	Interacts
Protein kinase C	Activates
Protein kinase D	Regulated by
Protein kinase, camp dependent	Activates
Protein kinase, DNA-activated, catalytic polypeptide	Binds
Protein phosphatase 1	Through Cdk9 phosphorylation
Protein phosphatase 1 regulatory inhibitor subunit 8 isoform alpha	Binds
Protein phosphatase 1, catalytic subunits	Binds
Protein phosphatase 2, catalytic subunits	Modulated by
Protein phosphatase 3, catalytic subunits	Activates
Protein tyrosine phosphatase, nonreceptor type 23	Upregulates
Protooncogene tyrosine-protein kinase SRC	Activates
PTK2 protein tyrosine kinase 2 isoform a	Activates
PTK2B protein tyrosine kinase 2 beta isoform a	Induces phosphorylation
Purine-rich element-binding protein A	Binds
RAD51 homolog protein isoform 1	Interacts
RAN-binding protein 5	Binds
Ras homolog gene family, member A	Activates
Ras-related C3 botulinum toxin substrate 1 isoform Rac1	Activates
Ras-related C3 botulinum toxin substrate 2	Activates
RD RNA-binding protein	Associates
RelB	Through inhibition of tnf*α*
Replication factor C2 (40 kDa) isoform 1	Interacts
Replication factor C3 isoform 1	Interacts
Replication factor C4	Interacts
Replication factor C5 isoform 1	Interacts
Replication factor C large subunit	Interacts
Replication protein A1, 70 kDa	Interacts
Reticuloendotheliosis viral oncogene homolog B	Interacts
Retinoblastoma 1	Inhibited by
Retinoblastoma-like 2 (p130)	Binds
Rho GDP dissociation inhibitor (GDI) alpha	Downregulates
Ribosomal protein L3 isoform a	Interacts
RNA guanylyltransferase and 5^′^-phosphatase	Binds
RNA polymerase II, polypeptide H	Activates
S-phase kinase-associated protein 2 isoform 1	Enhanced by
Secretoglobin, family 2A, member 2	Upregulates
Selectin E precursor	Upregulates
Semaphorin 4D	Upregulates
SHC (Src homology 2 domain containing) transforming protein 1 isoform p52Shc	Induces phosphorylation of
Signal transducer and activator of transcription 1 isoform alpha	Upregulates
Signal transducer and activator of transcription 3 isoform 2	Activates
Signal transducer and activator of transcription 6	Interacts
Single-stranded DNA-binding protein 1	Upregulates
Sirtuin 1	Regulated by
SKI-interacting protein	Associates
SKIP	Interacts through c-Myc and Menin
Small GTPase protein E-Ras	Activates
Small inducible cytokine precursors	Inhibits
Small inducible cytokine subfamily E, member 1	Upregulates
Small nuclear ribonucleoprotein polypeptide	Stimulated by
Solute carrier family 22, member 1 isoform a	Regulated by
Solute carrier family 5 (sodium/glucose cotransporter), member 1	Inhibits
Solute carrier family 6 (neurotransmitter transporter, dopamine), member 3	Downregulates
Sp1 transcription factor	Interacts
SP110 nuclear body protein isoform a	Upregulates
Sp3 transcription factor isoform 1	Interacts
Sp4 transcription factor	Interacts
Spermidine/spermine N1 acetyltransferase	Upregulates
Splicing factor, arginine/serine-rich 1 isoform 1	Inhibits
Splicing factor, arginine/serine-rich 7	Inhibits
Squamous cell carcinoma antigen recognized by T cells 3	Regulated by
SRB7 suppressor of RNA polymerase B homolog	Interacts
Src homology 2 domain containing transforming protein C3	Induces phosphorylation
Succinate dehydrogenase complex, subunit B, iron sulfur (Ip)	Binds
Superoxide dismutase 1, soluble	Downregulates
Superoxide dismutase 3, extracellular precursor	Downregulates
Suppressor of Ty 4 homolog 1	Activated by
Suppressor of Ty 5 homolog	Stimulated by
SWI/SNF-related, matrix-associated, and actin-dependent regulator of chromatin	Binds
Syndecan 1–4 precursors	Binds
T-box 21	Upregulates
T-cell receptor zeta chain isoform 1 precursor	Interacts
TAF12 RNA polymerase II, TATA box-binding protein (TBP)-associated factor, 20 kDa	Interacts
TAF9 RNA polymerase II isoform a	Interacts
TAR DNA-binding protein	Inhibited by
TAR RNA-binding protein 1	Regulates
TAR RNA-binding protein 2 isoform a	Synergizes
TAT-interactive protein, 72 kDa	Binds
TATA box-binding protein	Interacts
TATA box-binding protein-associated factor 2F	Interacts
TBP-1 interacting protein isoform 2	Inhibited by
TBP-associated factors	Interacts
Telomerase reverse transcriptase isoform 1	Downregulates
Testis-specific histone H2B	Binds
TFIIA alpha, p55 isoform 1	Stabilizes
TH1-like protein	Associates
Thrombospondin 1 precursor	Binds
Thyroid hormone receptor, alpha isoform 2	Binds
Tight junction protein 2 (zona occludens 2) isoform 1	Downregulates
Tissue inhibitor of metalloproteinase 1 precursor	Interacts
Tissue inhibitor of metalloproteinase 2 precursor	Interacts
TNF receptor-associated factor 4	Inhibited by
Transcription elongation factor A (SII), 3	Interacts
Transcription elongation factor A1 isoform 1	Interacts
Transcription elongation factor A protein 2 isoform a	Interacts
Transcription elongation regulator 1 isoform 1	Associates with
Transcription factor 3	Regulated by
Transcription factor 4 isoform b	Inhibits
Transcription factor 7-like 2 (T-cell specific, HMG-box)	Regulated by
Transcription factor AP-4 (activating enhancer binding protein 4)	Regulated by
Transcription factor CP2	Inhibited by
Transforming growth factor, alpha	Upregulates
Transforming growth factor, beta 1	Inhibited by
Transforming growth factor, beta 2	Upregulates
Transporter 1, ATP-binding cassette, subfamily B	Upregulates
Transportin 1	Binds
Tripartite motif-containing 22	Inhibited by
TUBB4Q: tubulin, beta polypeptide 4, member Q	Downregulates
Tubulin alpha 6	Binds
Tubulin, alpha 1	Binds
Tubulin, alpha 1a	Binds
Tubulin, alpha 2 isoform 1	Binds
Tubulin, alpha 4	Binds
Tubulin, alpha 8	Binds
Tubulin, alpha, ubiquitous	Binds
Tubulin, beta	Downregulates
Tubulin, beta 2	Binds
Tubulin, beta 2B	Downregulates
Tubulin, beta 4	Binds
Tubulin, beta 6	Binds
Tubulin, beta 8	Binds
Tubulin, beta, 2	Binds
Tubulin, beta, 4	Binds
Tumor necrosis factor (ligand) superfamily, member 10	Inhibits
Tumor necrosis factor alpha	Interacts
Tumor necrosis factor receptors	Upregulates
Tumor protein p53	Binds
Tumor protein p73	Binds
Tyrosine hydroxylase isoform b	Downregulates
Ubiquitin B precursor	Ubiquitinated by
Ubiquitin C	Ubiquitinated by
Ubiquitin-activating enzyme E1	Ubiquitinated by
Ubiquitin-conjugating enzyme E2D 1	Ubiquitinated by
Upstream-binding protein 1 (LBP-1a)	Inhibits
Urokinase plasminogen activator preproprotein	Upregulates
V-akt murine thymoma viral oncogene homolog 2	Activates
V-akt murine thymoma viral oncogene homolog 3 isoform 1	Activates
V-fos FBJ murine osteosarcoma viral oncogene homolog	Interacts
V-Ha-ras Harvey rat sarcoma viral oncogene homolog isofrom 1	Activates
V-rel reticuloendotheliosis viral oncogene homolog	Interacts
Vascular cell adhesion molecule 1 isoform a precursor	Upregulates
Vascular endothelial growth factor A isoform b precursor	Cooperates with
Vitronectin precursor	Competes with
Wolf-Hirschhorn syndrome candidate 2 protein	Downregulates
YY1 transcription factor	Inhibited by
Zinc finger and BTB domain containing 7A	Binds

**Table 2:** Tat-based therapeutics.

Compound/class	Mechanism of action	Reference(s)
DRB	Purine nucleoside analog; inhibits cyclin-dependent kinases	[23]
Flavopiridol (flavonoids)	Inhibits cyclin-dependent kinases	[24]
Seliciclib	Inhibits cyclin-dependent kinases	[25]
2^′^-O-Methyl/LNA oligoribonucleotides	Binds TAR	[26]
Phosphodiester/phophothiote oligonucleotides	Binds TAR	[27]
PNA-(TAR-16)	Polyamide nucleotide analog; binds TAR	[28]
Acetylpromazine	Binds 5^′^ bulge of TAR	[29]
O,O^′^-Bismyristoyl thiamine disulfide	Inhibits nuclear translocation of Tan and NF-*κ*B, via interaction with cysteine region	[30]
Cyclic peptides	Mimics basic region and binds TAR	[31]
Tat 9-K-biotin peptide	Binds TAR	[32]
CGP64222	Peptoid/peptide similar to Tat-basic domain; binds TAR	[33]
CGP40336A (polyamine-acridine based)	Binds TAR	[34]
Aminoglycoside-arginine conjugates	Binds TAR in the major groove of the bulge and upper portion of the stem	[35]
Transdominant Tat mutants	Binds TAR	[36]
Biscationic diphenylfuran derivatives	Binds TAR	[35]
Neomycin (aminoglycoside)	Binds TAR, CXCR4, and other Tat targets	[37]
D-penicillamine	Binds Tat stably through cysteine residues	[38]
Stilbene (CGA137053)	Binds Tat directly	[39]
Suramin and derivatives	Competes with heparin/heparin sulfate for binding to the basic region of Tat; inhibits extracellular functions of Tat	[40]
Benzodiazepine derivatives	General inhibition of HIV-1 transcription and Tat transactivation	[41, 42]
Benzothiophene derivatives	—	[43]
Temacrazine (bistriazolonoacridones)	—	[44]
Fluoroquinolone derivatives	—	[45, 46]
